# Cause and Effect

**Published:** 1907-06

**Authors:** 


### EDITORIAL DEPARTMENT.

THIS ISSUE completes my twenty-second year. The "Red Back" will begin its twenty-third volume next month, and it enters upon the year with renewed courage, and determination to hold aloft, as it has steadfastly done nearly a quarter of a century, the banner of legitimate, ethical, scientific medicine, and defends the same against all assaults of the enemy, the world, the flesh and the devil. It is my nature to rise with opposition,—and knowing that the cause is righteous and must prevail, and backed and sustained by the continuous support of the cleanest and best men in the profession, I will strike so long as God gives me strength to wield a weapon. I renew my thanks to the Old Guard and bid them be "steady on the left."

### CAUSE AND EFFECT.

### THE RELATION OF ALCOHOL TO INSANITY.

How much longer will enlightened people deal with effects and ignore causes?

If mad dogs were at large giving people hydrophobia, would it be wiser to build costly asylums to care for the victims, at an expense of three-quarters of a million annually and rapidly and continually increasing, or to kill the dogs?

That is the situation in Texas, except that the victims transmit the poison "to the third and fourth generation," requiring more asylums and increased expense to care for the progeny.

### THE EFFECT.

The following figures are taken from a public address by Professor Marvin L. Graves, of the Medical Department, University of Texas, late Superintendent Southwest Texas Insane Asylum, at San Antonio, published in the TEXAS MEDICAL JOURNAL, April, 1905.

From 1860 to 1904 the population of Texas increased 504 per cent.

From 1860 to 1904 the insane in Texas increased 6800 per cent; or at a ratio of 13.7 to one of population.

In 1860 there were fifty insane persons in Texas, or one to 12.080 of population.

Now (1907) there are 5000 insane in Texas, or one to (about) every 600 population.

The first insane asylum was built (at Austin) in 1861. Fifty patients.

We now have four asylums—4500 patients.

Four years ago Governor Sayers said: "It is the proudest boast of my administration that there is not an insane person in Texas not cared for by the State." Now there are over 500 more than the asylums will accommodate—and they are in the jails. Chairman O'Neal at the recent Legislature tried to get an appropriation for more asylum room and failed; meantime these unfortunates are in jail, and the number is rapidly increasing.

The first appropriation for the insane (1861) was $12,000.

For the year ending August 31, 1904, the appropriation was $780,155 for the care of the insane.

The following figures are taken from the Comptroller's books:

Total appropriation for the four insane asylums of Texas, for 1904, $780,155; for 1906, $753,572; for 1907, $613,650; for 1908, $722,725. Grand total for support of the insane for five years, $3,539,285; yearly average, $707,857, nearly three-fourths of a million. Extend these figures at the ratio of the increase above, and in ten years we will probably have to have ten asylums to accommodate 10,000 insane at a cost of two and a half millions annually, or more.

THE CAUSE.

Now, what is the chief cause of the alarming increase of insane in Texas, and the tremendous increase of the burden of caring for them?

Dr. B. M. Worsham, Superintendent State Insane Asylum, Austin, Texas, writes me:

My Dear Doctor: Your letter of the 23d inst. has been received, asking me what percentage of insanity is due to alcohol. In reply I have to say that according to the best statistics, in all, 20 per cent of mental or nervous diseases, alcohol can be considered as a factor in causation. It is the consensus of opinion that fully 75 per cent of all cases of insanity is due to hereditary predisposition, 20 per cent to alcohol, leaving the other 5 for all other causes.

Very respectfully,

B. M. Worsham,

Superintendent.

Dr. Graves says: "The important factors in the production of insanity are heredity, alcoholism, in its various forms," and puts it at 60 per cent.

The following from Prof. T. D. Crothers, M. D., Professor of Nervous Diseases, Medical Department, University of New York, Superintendent Walnut Lodge Hospital, Hartford, Connecticut, and Editor-in-Chief of the *Journal of Inebriety,* the official organ of the American Society for the Study of Alcohol, will be of great interest in this connection:

HARTFORD, CONN., April 27, 1907.

MY DEAR DR. DANIEL: The enclosed items represent most conservative views on this subject. In my opinion they are very much underrated. But this is safe. In one of the New York asylums the superintendent found 70 per cent of the inmates who had either drank spirits to excess or were the children of excessive drinkers.

There is going to be a great awakening in this direction before long. Not on theory or the result of any individual opinion, but from facts that appeal to every business and scientific man. The whole alcoholic question is rapidly being settled from a commercial and scientific point of view.

The stupid chemist and physiologists who think that it is a laboratory problem and a question of statistics and shifting opinions are mistaken. To the business man, the employer of labor, and the financier alcohol is not a tonic or stimulant and can not be used in any proportions without periling their interests.

The saloon must be driven out, and there is no theory about this or speculation. The railroad companies and the insurance companies demand total abstinence and insist upon it.

No moderate drinking, no questions of the value of alcohol are considered by them. These are hard facts which are growing in every circle, and this is the side of the temperance question that is making tremendous strives.

Command me if I can be of any service, and believe me,

Yours truly,

T. D. CROTHERS.

Dr. Crothers enclosed in his letter the following:

"In 1890 the late Dr. Norman Kerr, of London, reported from an exhaustive study of the lunacy statistics of Great Britain that at least 40 per cent of all lunatics were both directly and indirectly caused by alcohol. A number of hospitals for the insane made new studies on the subject and concluded that from 10 per cent to 25 per cent of all the inmates were insane from the effects of alcohol. Also that a percentage unknown and not easily traceable were due to the same causes. A study was made of the lunacy statistics of France by Dr. Lagrand, of Paris, and his conclusion was that at least 50 per cent of all insanity, idiocy and epilepsy was due to the excessive use of alcohol. Dr. Clouston, of Scotland, one of the most eminent authorities in England, declares that 30 per cent of all the insane in England is due to this cause, and can be directly traced to it. He also asserts that alcohol is responsible for at least one-half of all the feeble-minded, insane and idiots of England, and that it is one of the most powerful causes of degeneracy. The latest statement on this subject is Dr. Savage in the Lumlein Lectures on insanity, delivered this year in London. He declares that alcohlic excess is one of the most prominent causes of insanity, and this belief is confirmed by statistics to which there can be no exceptions. In this country all studies indicate from 10 per cent to 20 per cent of the insane confined as due directly to alcohol. These are causes where the history is beyond all doubt. More careful observation indicates a much larger percentage. In my opinion a great many cases of insanity are put down as due to other causes. In deference to public opinion and as matter of pride, in much the same way as statistics of pneumonia are gathered. In a little town I found from a study of a death rate that 70 per cent occurred in persons who had used spirits to excess, and yet no mention was made of this in the study of the causes. The same is true of insanity. Per cent no doubt in the neighborhood of 50 or more could be traced to this cause. As you well know, we do not call things by the right name, and the tendency everywhere is to cover up anything that are called vices. Rev. Dr. Hale's remark that if they would banish the saloon and the use of alcohol as a beverage his church would guarantee to take care of all the paupers in the city of Boston, and do it well, is true in a larger sense than we understand at present."

I would like to give some authentic figures showing the relation of alcohol to crime, but the limits of this paper will not permit more than a mention. In Europe and America, from 50 per cent to 83 per cent of crime (I do not mean misdemeanors) is attributed to drink. And of capital offenses, fully 50 per cent. (Figures taken from report of International Penitentiary Congress, at Brussels, in 1900.)

Enough has been stated, I think, to show that drink is responsible for more crime, insanity, poverty, misery and death than any other cause, and is the most potent factor in the swift decay of the human race! Do you know why scripture limits the descent of the

"sins of the father" to the "third and fourth generation?" It is a fact in science. Hear the great naturalist, Darwin: "The families of drunkards do not descend beyond the fourth generation." And Morel (the great French authority) says: * * * "In the first generation there are moral depravity and alcoholic excesses. In the second, drunkenness and maniacal outbursts. In the third, melancholia, hypochondria and impulsive ideas, particularly those of murder. In the fourth the imbecile and idiot appear, and the family becomes extinct." (Race suicide.)

### THE REMEDY—REMOVE THE CAUSE.

Appeal to the courts to test the constitutionality of the liquor license law.

Judge Artman, of Boone county, Indiana (*Literary Digest*, April 6, 1907) has held it to be unconstitutional, and the case has gone to the Supreme Court.

"Quoting a sheaf of decisions from the United States Supreme Court and various State Supreme Courts in which the evils and miseries due to strong drink are dwelt upon, and quoting a decision of the Indiana Supreme Court declaring a law permitting prize fighting unconstitutional because it was 'opposed to the spirit of the Constitution,' Judge Artman maintained that the evil influences of prize fighting 'are insignificant when compared with the destructive results of the liquor traffic,' and he, therefore, declared:

"In view of these holdings, based, as they certainly are, upon good reason and sound common sense, it must be held that the State can not, under the guise of a license, delegate to the saloon business a legal existence, because, to hold that it can, is to hold that the State may sell and delegate the right to make widows and orphans, the right to break up homes, the right to create misery and crime, the right to make murderers, the right to produce idiots and lunatics, the right to fill orphanges, poorhouses, insane asylums, jails and penitentiaries, and the right to furnish subjects for the hangman's gallows.

"With due appreciation of the responsibilities of the occasion, conscious of my obligations under my oath to Almighty God and to my fellow man, I can not by a judgment of this court authorize the granting of a saloon license, and the demurrer to the amended remonstrance is therefore, overruled, the amended remonstrance is sustained, and the application is dismissed at the cost of the applicant."

The logic that prohibits the sale of morphine and cocaine should justify the prohibition of the indiscriminate sale of a worse and more destructive poison. The arguments that justify the saloon would apply as well to opium smoking joints and the indiscriminate sale of morphine and cocaine.

Alcohol as a remedy is not indispensable in the practice of medicine.

ALCOHOL AND TUBERCULOSIS.

There are one hundred and fifty thousand deaths from consumption, annually, in America; four hundred and eleven daily; one every three minutes. On the best authority it can be shown that alcohol, directly, and indirectly by the transmitted craving for drink, the hereditary neurasthenia and unstable constitution, is a large factor in the causation of consumption, and larger still in the mortality.

For a century or more it was the universal practice with the best physicians to treat consumption with whisky and brandy, egg-nogg being the favorite prescription, under the false belief that it was a "tonic," a "stimulant," and that it "assisted nature" to throw off the disease.

It is now known (and I make the statement on the authority of the great Metchnikoff; Harben Lectures) that it is neither tonic nor stimulant, but a posion to, and paralysant of, the phagocytes and the "opsonins," nature's agents for the elimination of pathogenic bacteria; and, consequently, the administration of either alcohol and laudanum, by putting these agents to sleep—defeats and not assists—nature. This can be verified, and has repeatedly been verified, under the microscope. Hence it will readily be understood why alcoholics are more prone to infectious diseases and yield more readily to attack than abstainers, a fact observed many years; and why consumption is especially fatal amongst brewers and beer drinkers.

THE TEMPERANCE MOVEMENT.

The medical profession all over the world is fast awakening to a realization of the fact that alcohol is the great enemy of mankind, and that they have been using it as a remedy on false assumptions, or, rather, in ignorance of its real action in disease.

The British Medical Association established some years ago the custom of having Temperance Breakfasts during the annual meetings, at which the subject was discussed by the ablest members. Following this laudable example, the American Society for the Study of Alcohol has inaugurated Temperance Lunches during each annual session of the American Medical Association,—elaborate functions, minus wine; and it is most gratifying to note the active interest that is taken by the leading men of the great A. M. A.—the largest body of medical men in the world—in this important movement. At the June session of that body at Atlantic City such a "lunch" was served, Dr. H. O. Marcy, of Boston, presiding, and the entire membership—some several thousand—were invited to be present, together with their visiting ladies. How many were present, I do not know. Addresses—informal—were made by Dr. Marcy and by the President, Jos. D. Bryant; the retiring President, Mayo; ex-Presidents Jno. A. Wyeth, Matthews, Musser, Shoemaker, Reed, and other celebrities. It is also gratifying to note the active interest the general government is taking in the temperance reform movement, the President having appointd the Surgeon of the Army, the Surgeon Inspector of the Navy, Prof. W. T. McNicholl, of New York, and Prof. T. D. Crothers, of Hartford, delegates to the eleventh International Anti-Alcohol Congress, to be held in Stockholm, Sweden, July 28th, prox.

Th temperance question is not one of religion,—but of social and political economy; it is a question of public health and of race integrity.

The entire revenue from State liquor license in Texas is said to be $600,000 annually. It has been shown elsewhere (cause and effect) that the cost of caring for the insane, alone,—50 per cent or more of whom are said to be the victims of alcohol,—exceeds this amount by more than 20 per cent; or, to be exact, by $107,000 annually; while Hon. T. W. Gregory, of Austin, recently showed in a public address that the entire revenue to the State from liquor licenses is not sufficient to pay the cost of enforcing the criminal laws, in the arrest and prosecution of murderers, more than one-half of whom are victims of drink.

The worst feature of the alcohol evil is the "saloon." The saloon is made attractive,—seductive,—by brilliant lights, warmth in winter, pictures, flowers, music, and the privilege of dominoes or rolly bolly for the drinks. The high tax the government puts on liquor drives the unscrupulous retail dealer to dilute it to cheapen it, and to adulterate it to make it get in its delirium-producing effects; and, as dispensed over most bars, it is absolutely poisonous. It is asserted as a fact that it is "doctored" with

*coculus indica,* copperas, fusil oil,—and even with wood alcohol, a poison that is fatal in large doses, and destructive to eye-sight in small and repeated doses.

Advancing civilization, and every consideration of humanity and of race integrity, demand the eradication of this intolerable, death and destruction-dealing evil.

---

The Deadly Fourth of July Celebration is upon us. I hereby issue the "Red Back's" annual warning to doctors and parents to suppress the death-dealing toy pistol, and the deadly firecrackers. It is unnecessary to again give the statistics of lockjaw and death from these causes, or to again point out that, for some reason not known, wounds from these things, however slight, are apt to produce lockjaw, and rarely every case, if not every one, proves fatal. Readers must warn the people to suppress this evil. Every town and city should prohibit the sale of fireworks of all kind, and of the terrible "toy pistol" especially. Johnnie must be educated in the elements of noise-making by some less dangerous means.

